# Identifying predictive motor factors for falls in post-menopausal breast cancer survivors

**DOI:** 10.1371/journal.pone.0173970

**Published:** 2017-03-17

**Authors:** Marek Zak, Malgorzata Biskup, Pawel Macek, Halina Krol, Szymon Krupnik, Anna Opuchlik

**Affiliations:** 1 Department of Physical Rehabilitation in Rheumatology and Geriatrics, University School of Physical Education, Krakow, Poland; 2 Department of Rehabilitation, Holycross Cancer Centre, Kielce, Poland; 3 Faculty of Health Sciences, The Jan Kochanowski University, Kielce, Poland; 4 Department of Epidemiology and Cancer Control, Holycross Cancer Centre, Kielce, Poland; 5 Faculty of Medical Sciences, School of Economics, Law and Medical Sciences, Kielce, Poland; West Virginia University, UNITED STATES

## Abstract

**Objective:**

Breast cancer treatment, including radical surgery, is also pursued as late as the 7th - 8th decade of women’s lives. Standard physical rehabilitation procedures offered to those women are predominantly focused on attenuating specific functional deficits of the upper limb and trunk. Seldom do they entail any regimens specifically aimed at recovering overall functionality, and reducing exposure to falls-risk.

The study aimed to assess potential interrelationships between the self-reported falls, individual functional capabilities and appreciably reducing exposure to falls-risk in a group of post-menopausal, post-surgical breast cancer survivors.

**Methods:**

The study recruited 102 women (aged 65–79; mean age 70.2), post-surgical breast cancer survivors. The subjects were stratified by age into three groups: Group 1 (65–69 years); Group 2 (70–74 years), and Group 3 (75–79 years). Individual functional capabilities were assessed with Eight-foot up & go test (8UG), chair stand test (CST), and 2-minute step test (2ST). Tinetti POMA test was applied to assess gait and balance disorders. Self-reported falls in the past year were ascertained through a questionnaire.

**Results:**

Assessment of individual aerobic endurance (2ST) also demonstrated a clear deficit in the mean scores category in all respective age sub-groups, as compared against the reference values. The deficits ranged from 4.86 to 15.90 steps less than the normative values; the oldest subjects demonstrating the largest deficit. The aerobic endurance tests results significantly impacted the ultimate assessment of an individual falls-risk in the oldest group. The analysis of the number of falls sustained within the recent year indicated that 43.67% of the subjects fell victim of such incidents.

**Conclusion:**

An individual exposure to falls-risk was found to be appreciably more dependent upon individual aerobic endurance rather than overall strength of the lower part of the body in the breast cancer survivors over 75.

## Introduction

A sustained rise in the incidence of breast cancer has sparked off some concerns that during comprehensive therapeutic management, including radical surgical intervention, there is an appreciable reduction in physical activity and functional capabilities in the older women.

Effective breast cancer treatment and attendant surgical interventions are sometimes pursued as late as the 7th - 8th decade of their lives. Most of them boast an appreciable success rate, consequently facilitating complete recovery and thus allowing senior women to get on with their lives despite having gone through such a traumatic experience.

Physical rehabilitation procedures offered to these senior post-surgical breast cancer survivors are generally focused on attenuating the limb edema on the operated side, improving overall range of motion and muscle strength within the limb, and alleviating any residual dysfunctions around the trunk area [1]. Very seldom indeed do any such rehabilitation programs comprise any physical regimens specifically aimed at appreciably improving their overall functional capabilities (balance and gait), and reducing exposure to falls-risk and any potentially adverse consequences of falls.

Furthermore,. approx. 50% of working age women under treatment for breast cancer are not involved in any form of intense physical activity (defined as > 6MET-h per week), whereas for 25% of them this activity accounts for less than 3 MET-h, which is equivalent to walking at the speed of 4.8 km/h [2,3].

It should also be noted that a steadily increasing proportion of women diagnosed with breast cancer is expected to be at an advanced age [4,5]. We can therefore well expect an increase in the number of women after mastectomy pursuing predominantly sedentary lifestyle.

Advanced age compounded by the side effects originating from a complex therapeutic management of cancer makes the initiation and uptake of remedial measures to maintain function more difficult, but of crucial importance in secondary preventive care, with a view to maintaining individual self-reliance and quality of life.

Female gender is deemed a legitimate risk factor for balance disorders and accidental falls also by Francis and Rubenstein [6,7]. Women sustain falls three times more frequently and are twice as likely to suffer resultant bone fractures. Consequently, they are also hospitalized five times more often [8]. It might be appropriate to highlight at this juncture that commonly acknowledged falls-risk factors are the following: age > 75 years, > 5% loss of muscle strength per year, lower productivity of circulatory system expressed by a decrease of 1m/kg/min, routine intake of > 5 medications per day, and a walking speed < 0.08 m/s.

There is precious little to be found in the literature on falls, falls-risk, functional capability, or balance in the older women undergoing breast cancer treatment. As falls pose a huge threat to individual self-reliance, quality of life and psychological wellbeing, it is essential to ascertain whether falls are common in women undergoing breast cancer treatment, and which specific functional capabilities may actually need improvement through intervention to reduce their exposure to falls-risk [9,10].

The present study aims to provide some brand-new insights into the functional capabilities (balance, agility and gait) of senior breast cancer survivors and their exposure to falls-risk, while in view of its application character may also offer some hands-on guidance to the rehabilitation teams working with this group of patients, with a view to giving due consideration to the specific rehab procedures aimed specifically at enhancing overall functional capabilities (balance and gait), while designing and structuring any individually tailored rehab regimens.

## Methods

The study group was comprised of women over 65 years of age, having undergone a surgical intervention for breast cancer in the Cancer Centre. The subjects had sustained a radical mastectomy on one side of the chest, without the benefit of a reconstructive surgery. Written invitations to take part in the survey had been sent out to all the women. Out of 466 post-surgery women 48 died (either in the hospital or elsewhere), 8 women did not consent to participating in the study (7 due to poor health, 1 due to lack of time), and 237 failed to respond to the invitations.

The study recruited 102 (21.8% of those actually approached) women aged 65–79 (mean age 70.2 years; Me = 70 years, SD = 4.33 years) undergoing breast cancer treatment at the Cancer Centre. The subjects were divided up into the three age groups: Group 1 (65–69 years of age; N = 50; 49%); Group 2 (70–74 years of age; N = 31; 30.4%), and Group 3 (75–79 years of age; N = 21; 20.6%).

The gait and balance disorders were assessed with the Tinetti POMA test [11]. The data on self-reported falls were collected by means of a questionnaire, i.e. inquiry as to the actual number of falls sustained within the last 12 months immediately preceding the present study.

Gait speed was ascertained on a 6-metre distance, in line with the formula: gait speed = distance covered: time taken to cover the set distance.

Their balance agility and gait were assessed using the Eight-foot up & go (8UG) test (assessment of individual agility and balance, while moving), a 2-minute step test (2ST) (assessment of aerobic endurance), and a chair stand test (CST) (assessment of the strength of the lower part of the body), i.e. three of the component tests in the Senior Fitness Test battery [12].

All study protocol procedures were duly approved by the Bioethics Review Committee, District Chamber of Physicians (No 19/KBL/OIL/2011).

Table 1 shows the baseline characteristics of the patients (study subjects).

**Table 1:** Baseline characteristics.

Variable	N = 102
**Demographic**	
Age (years), Mean, SD	70.2 (4.33)
Body weight (kg) Mean, SD	71.13 (12.21)
Body height (cm) Mean, SD	161.23 (5.54)
Body mass index (kg/m^2^) Mean, SD	27.33 (4.28)
**Education**	
Primary (%)	28.9
Secondary (%)	40.5
University (%)	16.8
Other (vocational training only) (%)	13.8
**Marital status**	
Married (%)	54.3
Unmarried (%)	4.6
Divorced (%)	5.3
Widowed (%)	35.8
**Dominant side**	
Left (%)	6.4
Right (%)	93.6
**Operated side**	
Non dominant	53.2
Dominant	46.8
**Adjuvant therapy**	
Radiotherapy (%)	1.2
Chemotherapy (%)	6.9
Hormonal therapy (%)	26.6
Radiotherapy + Chemotherapy (%)	23.1
Radiotherapy + Hormonal therapy (%)	4.1
Chemotherapy + Hormonal therapy (%)	12.1
Radiotherapy + Chemotherapy + Hormonal therapy (%)	26
**Falls**	
65–69 years old (%) no falls/1 fall/>1 fall	68/20/12
70–74 years old (%) no falls/1 fall/>1 fall	48/29/23
75–79 years old (%) no falls/1 fall/>1 fall	43/33/24

SD—Standard Deviation.

### Statistical analysis

All examination results were analyzed using Med. Calc 12.1.0. and p≤0.05 were considered statistically significant. The following methods were applied in the statistical analyses: The Kolmogorov-Smirnov compatibility test for the assessment of normal distribution of the tested variables. Basic statistics (i.e. arithmetic mean, standard deviation, median, extreme values) to provide a general characteristics of the study subjects. The Kruskal-Wallis one-way analysis of variance by the ranks test for the comparison of variations of parameters in the studied group of subjects under the influence of independent variables of abnormal distribution, along with a post hoc Conover test for multiple comparisons of the mean values of the assessed parameters. The Mann-Whitney test for the comparison of the mean values of the assessed parameters, as well as the differences between the respective parameters within the group of subjects under study. The assessment of the mean trends and of the diffusion, with a view to comparing the values of the assessed parameters with the reference values. The correlation analysis for assessing the significance of the correlation between the respective parametric variables. In order to assess the incidence of falls within the recent year, as well as the final scores of the Tinetti POMA test (i.e. the cut-off point for the risk of falls was adopted at ≤19 points, and for the non-risk of fall at >19 points), the actual number of such occurrences within the study population was calculated.

## Results

Assessment of individual agility and dynamic balance when walking, as facilitated by the “Eight-foot up & go” test scores, demonstrated that the study subjects exceeded the set reference values for all respective age categories [12].

Assessment of the strength of the lower part of the body (“Chair stand test” scores) demonstrated a clear deficit in the mean scores category in all respective age sub-groups of women, as compared against the reference values. The deficits ranged from 0.32 to 1.24 chair rises less than the normative values would suggest, and the oldest appeared to have the largest deficit.

The assessment of individual aerobic endurance (“2-minute step test” scores) also demonstrated a clear deficit in the mean scores category in all respective age sub-groups of women, as compared against the reference values. The deficits ranged from 4.86 to 15.90 steps less than the normative values would suggest, and the oldest appeared to have the largest deficit.

Analysis of the mean values of the variable under study in the *post hoc* test scores revealed a statistically significant difference between the respective sub-group subjects (Sub-group 1, 2 and 3) (p<0.05) (Table 2).

**Table 2:** Functional capabilities test results stratified by age, juxtaposed against normative values in the elderly women.

**“Eight-foot up & go” test**							**Reference values**		***p***
**Group**	**N**	**Age group (years)**	**Mean**		**SD**		**Min**	**Max**	
1	50	65–69	7.70		2.30		4.80	6.40	**0.028**
2	31	70–74	9.94		4.34		4.90	7.10	**0.000**
3	21	75–79	11.30		4.52		5.20	7.40	**0.000**
**„Chair stand test”**							**Reference values**		
**Group**	**N**	**Age group (years)**	**Mean**		**SD**		**Min**	**Max**	
1	50	65–69	10.44		2.67		11	16	**0.000**
2	31	70–74	9.68		3.89		10	15	**0.000**
3	21	75–79	8.76		1.92		10	15	**0.000**
**„2-minute step test”**						**Reference values**			
**Group**	**N**	**Age group (years)**	**Mean**	**SD**		**Min**		**Max**	
1	50	65–69	68.14	16.38		73		107	**0.000**
2	31	70–74	59.00	15.55		68		101	**0.000**
3	21	75–79	52.10	19.28		68		100	**0.000**

SD–Standard Deviation, Min–minimal value of variable, Max–maximal value of variable.

There was a statistically significant age effect on the gait speed in all respective age sub-groups of women (p<0.05) (Table 3).

**Table 3:** Assessment of the age factor impact on the gait speed in all respective age sub-groups of women.

Study group (N)	Age group	Mean	SD	Median	p–value	Discriminatory group
1 (50)	65–69	0.69	0.19	0.67	**p = 0.0002**	(2)(3)
2 (31)	70–74	0.55	0.16	0.60		(1)
3 (21)	75–79	0.48	0.17	0.49		(1)

Based on the review of the final Tinetti POMA test results, conducted in all respective age sub-groups, the study population was subsequently divided into the lower and higher risk groups in terms of respective propensity for accidental falls. Nearly 10% of the over 70 study subjects were classified as remaining at high falls-risk (Table 4).

**Table 4:** Distribution into the respective risk groups, based on the final Tinetti POMA test scores.

Study group (N)	Age group (years)	Lower risk group (number/%)	Higher risk group (number/%)
1 (50)	65–69	49 (98%)	1 (2%)
2 (31)	70–74	27 (87%)	4 (13%)
3 (21)	75–79	18 (86%)	3 (14%)

The analysis of the number of falls sustained within the recent year indicated that 43.67% of the subjects fell victim of such an experience.

There was a statistically significant relationship between gait speed and a risk of fall (p<0.05). In all respective age sub-groups a higher gait speed was found to significantly diminish that risk (Table 5).

**Table 5:** Correlations between the respective Tinetti POMA test final scores in all respective age sub-groups of women.

Test	Study group (N)	Correlation factor (r)	p-value
**Gait speed**	1 (50)	0.5070	**p<0.0002**
	2 (31)	0.6080	**p = 0.0003**
	3 (21)	0.5935	**p = 0.0046**
**„Chair stand test”**	1 (50)	0.5736	**p<0.0001**
	2 (31)	0.6512	**p = 0.0001**
	3 (21)	0.3268	p = 0.1482
**„2-minute test”**	1 (50)	0.1524	p = 0.2907
	2 (31)	0.2994	p = 0.1017
	3 (21)	**0.4875**	**p = 0.0250**

In two out of three groups under study, the test scores (Tinetti POMA test) pertaining to the strength of the lower part of the body significantly impacted an individual risk of fall (Table 5).

The results yielded by the aerobic endurance tests significantly impacted the ultimate assessment of an individual falls-risk in the oldest group only (Table 5).

Statistically significant differences were noted in the gait speed between the women who had been categorized (based on the final Tinetti POMA test scores) as at high risk (0–18) and low risk (19–28), respectively. The lower falls-risk sub-group (Median 0.61 m/s), and the higher risk sub-group (Median 0.29 m/s) were significantly different (p<0.001).

Significant differences in the strength of the lower part of the body between the women who had been allocated to the lower falls-risk sub-group and the ones from the higher risk sub-group were observed (p<0.05).

## Discussion

Individual functional capability is widely acknowledged to be of vital importance for older people in terms of coping effectively with the daily tasks and everyday challenges posed by a diversity of environmental factors (uneven floors, stairs, etc.). Older people are routinely faced with numerous obstacles that act as the specific barriers for participation in everyday activities, including the lack of, or incorrect information provided by the health care professionals. Adequate and appropriate encouragement offered to a patient upon discharge from hospital is pivotal for the effective uptake of a physical rehabilitation program [13].

In the older women, following breast cancer treatment, there is a deficit of endurance and lower body strength (compared to published normative data for the same age groups) and a higher falls-risk in those with poor balance. As seen in healthy older women, there is a decline in functional capabilities with increasing age, unlikely to be resultant from treatment [12,14].

Numerous authors emphasize that breast cancer treatment results in a diversity of side effects, which may hinder physical activity and reduce the uptake of exercise interventions [15,16,17]. It has been established, for instance, that by far the greatest limitation of physical activity is encountered in the women with highly malignant tumours (> = G3). During the adjuvant therapy, in the subjects who have undergone chemotherapy and radiotherapy physical activity tends to decrease nearly 3-fold, i.e. from 36 MET-hours/week down to 15 MET-hours/week, as compared to the ones who had not been subjected to it, or underwent a hormone therapy instead.

Limited physical capability is particularly apparent in decreased maximum oxygen consumption rate (VO2max), and in other symptoms referenced in the functional examinations, or questionnaires comparing physical activity of the subjects before and after the implementation of the actual treatment management [18,19].

Among the other reported causes of diminished physical performance are pain, edema, restricted body movement and muscular weakness which lead to diminished overall physical activity and a lifestyle virtually devoid of significant physical effort [20].

Diminished physical performance manifested itself as an appreciably faster sub-maximal heart rate, as well as by twice the rate of oxygen consumption at the same energy equivalent. Fatigue appears faster, consequently causing the body’s center of gravity to sway sideways more, reaction time increases, muscle strength in the lower extremities diminishes, and all those factors combined stand for an increased falls-risk.

Interestingly, no differences in the level of physical performance were dependent on the breast cancer treatment method (surgical treatment and surgical treatment accompanied by chemotherapy and radiotherapy) suggesting that restricted physical activity and movement were the main contributing factors [21].

In view of the above, it seems both prudent and justifiable to introduce a range of tests aimed at assessing individual functional capabilities in the women treated for breast cancer, initially prior to a radical surgical intervention, and then within certain intervals following its completion (e.g. every few months), simultaneously putting the subjects on an individually tailored rehabilitation exercise regimen expected to be of overall advantage to them.

In the present study we also assessed an individual’s propensity for falls using the Tinetti POMA test and a self-report comprised within a falls questionnaire. The results revealed that 10% (9.7%) of women under breast cancer treatment happened to be in the high risk of fall group. Indeed, 43.67% of the subjects reported a single, or recurrent falls sustained within the recent year. The number of falls increased with age. In the women following breast cancer treatment, a fall constitutes a significant health hazard, not only due to the risk of fracture, but also to a diversity of potential complications that might originate in the upper limb on its operated side, e.g. edema.

In the breast cancer survivors (BCSs) the risk of bone fracture is significantly higher (despite no bone metastases) than in the non-BCSs within the same age group. Radiotherapy effectively changes the bone density and consequently its micro-architecture [22]. The reduction of bone density reduces physical activity, while a decrease in physical activity increases individual exposure to falls-risk. Besides, over 50% of women study subjects had undergone radiotherapy as an adjuvant therapy. However, the percentage of fallers in these women following cancer treatment was not markedly different form the general older population [23].

Winters-Stone et al. also assessed the women (n = 59) undergoing breast cancer treatment, with a view to establishing their propensity for falls. The subjects were queried about the number of accidental falls actually sustained within the recent year. The findings implied that the recently treated postmenopausal BCSs sustained a higher number of falls, as compared to the population averages for the community-dwelling elderly adults. The authors reported that 58% of the BCSs had sustained a fall within the year preceding the actual enrolment into the study, whereas nearly half of them (47%) fell victim of such an experience within 6 months of its commencement [23].

The findings further implied that the balance-related problems may have been related to the specific changes in the vestibular system, associated with the chemotherapy treatment [23].

The findings of the above referenced study correlate with the results obtained by Chen et al., where the BCSs were exposed to a 15% higher falls-risk than the non-BCSs [24].

Significant changes in posture stability were also observed by Wampler et al. in the study embracing 22 women undergoing taxane chemotherapy, as compared to the non-BSCs control group. The authors noted that the BSCs treated with the taxane-based chemotherapy had worse visual acuity at the low, although not at the high contrast, as compared to the controls [25].

The studies of Winters-Stone, as well as those by Wampler, imply that breast cancer treatment may adversely affect the vestibular impulse to the balance control, which consequently may precipitate accidental falls in the subjects [23,25].

Similar symptoms are reported by the patients undergoing chemotherapy treatment. Based on the study of the BCSs still undergoing chemotherapy, and those who had this treatment already completed, Huang et al. concluded that those subjects experienced greater balance problems, as compared to the reference values [26].

The findings of the present study highlight the statistically significant correlations between gait speed (Groups 1, 2 and 3), strength of the lower part of the body (Groups 1 and 2), individual aerobic endurance (Group 3), and the results of the Tinetti POMA test which indicate appreciably better individual functional capability results in the higher scores in the Tinetti POMA test. The women subjects allocated to the lower risk group scored better in the gait speed, and the strength of the lower part of the body, as compared to those allocated to the high risk group.

There was a small percentage of women in the 'high falls-risk' category, as established with the aid of the Tinetti/POMA test, yet a much higher number/proportion of women who actually reported sustaining falls. Lower scores in the 2-minute step test might well be related to balance disorder encountered in post-mastectomy women.

Cancer disease and its therapeutic management leads to an appreciably diminished individual physical capacity, reduced functional capabilities, and a propensity to fall in these patients (be that still under therapeutic management, or already well into a post-intervention follow-up period). These effects are additive to the effects of age on functional capacities [12].Therefore, effective rehabilitation regimen specifically aimed at improving functional outcomes is vital for all cancer survivors.

## In conclusion

Key falls-risk factors, as commonly acknowledged throughout the literature on the subject, indicate that it is in fact the muscle weakness that is being deemed a clearly predictive variable for accidental falls in the seniors over 75. More than a quarter of the study subjects had undergone radio- and chemotherapy. Consequently, if radiotherapy should weaken cancellous (spongy) bone structure, thus negatively affecting overall physical activity, then reduced visual acuity caused by chemotherapy may well cause all physical activity to be shunned by a patient. This tendency is particularly manifest when physical activity of women subjects succumbs to linear regression with advancing age, i.e. as of the actual date of an adverse diagnosis, as attested to by a 2-fold decrease in the baseline values within a decade.

In the women breast cancer survivors over 75, however, exposure to falls-risk has been found to be far more dependent upon individual aerobic endurance than overall strength of the lower part of the body. Consequently, the principal focus in designing specific rehab regimens for this group of patients should rest firmly on boosting individual aerobic endurance, with the muscle strength being an issue of secondary therapeutic importance.

Walking speed changed with the patients’ age and incrementally diminished by as much as ca. 30% every 10 years. As such observations actually highlighted this as a complex rehabilitation issue, all attending physiotherapists should feel well motivated to have their therapeutic regimens, as offered to this particular age group, tailored accordingly, in due consideration of the present study findings.
